# Whole genome sequencing of low pathogenicity avian influenza virus (H6N2) detected from a Brazilian teal (*Amazonnetta brasiliensis*) in Brazil, 2023

**DOI:** 10.1128/mra.00158-24

**Published:** 2024-06-11

**Authors:** Andreina de Carvalho Araujo, Andrew Yong Cho, Laura Morais Nascimento Silva, Dagoberto Port, Meriane Demoliner, Juliana Schons Gularte, Micheli Filippi, Vyctoria Malayhka de Abreu Goes Pereira, Fabiane Fisch, Soledad Palameta, Jansen de Araújo, Joaquim Olinto Branco, Edison L. Durigon, Clarice Weis Arns, Sun-Hak Lee, Fernando Rosado Spilki, Dong-Hun Lee, Helena Lage Ferreira

**Affiliations:** 1 Department of Veterinary Medicine, FZEA-USP, University of Sao Paulo, Pirassununga, Sao Paulo, Brazil; 2 Avian Disease Laboratory, College of Veterinary Medicine, Konkuk University, Seoul, South Korea; 3 Graduate Program in Experimental Epidemiology Applied to Zoonoses, Veterinary Medicine and Animal Science School, University of São Paulo, São Paulo, São Paulo, Brazil; 4 Brusque City Hall - Municipal Department of Education, Brusque, Santa Catarina, Brazil; 5 Molecular Microbiology Laboratory, Department of Virology, Feevale University, Novo Hamburgo, Rio Grande do Sul, Brazil; 6 Polytechnic School, University of Vale do Itajaí, Itajaí, Santa Catarina, Brazil; 7 BSL-3 Laboratory of Virology and Applied Biotechnology, Department of Genetics, Evolution and Bioagents, Institute of Biology, University of Campinas – UNICAMP, Campinas, São Paulo, Brazil; 8 BSL3+ Laboratory of Clinical and Molecular Virology, Institute of Biomedical Sciences, University of São Paulo, São Paulo, São Paulo, Brazil; 9 Institut Pasteur of São Paulo/USP, University of São Paulo, São Paulo, São Paulo, Brazil; 10 Wildlife Health Laboratory, College of Veterinary Medicine, Konkuk University, Seoul, South Korea; 11 Konkuk University Zoonotic Diseases Research Center, College of Veterinary Medicine, Konkuk University, Seoul, South Korea; Portland State University, Portland, Oregon, USA

**Keywords:** wild birds, influenza, avian viruses, phylogenetic analysis

## Abstract

The whole genome sequence of a low pathogenicity avian influenza virus (H6N2) was sequenced from a Brazilian teal (*Amazonetta brasiliensis*) in Brazil, 2023. Phylogenetic analysis of the whole genome revealed a distinct genome pertaining to South American LPAIV from 2014 to 2016, indicating extensive circulation among South American wild birds.

## ANNOUNCEMENT

Migratory wild aquatic birds are natural reservoirs of avian influenza viruses (AIVs). Their migratory flyways can serve as routes for the spread of the virus across countries and continents (1). Several studies have reported South or North American lineage AIVs in Brazil, Peru, and Colombia (2–8) with reassortant AIVs between South and North American viruses in Chile and Argentina (9, 10). Nevertheless, studies on the ecology and epidemiology of AIV in South America have been limited. Here, we detected and sequenced the whole genome of a low pathogenic AIV (LPAIV) subtype H6N2 virus from a Brazilian teal (*Amazonetta brasiliensis*), resident bird in South America during an AIV surveillance in Brazil. We also conducted a phylogenetic analysis to trace the origin of the virus sequenced in this study.

Cloacal swab samples were collected in Santa Catarina state from 11 asymptomatic birds between 21 April and 22 April 2023, which belonged to five different species: *Amazonetta brasiliensis*, *Cairina moschata*, *Vanellus chilensis, Jacana jacana*, and *Gallinula galeata*. Samples from three *A. brasiliensis* and one *C. moschata* species tested AIV positive by the RT-qPCR test using the AIV matrix gene as the target (11), with Cycle threshold (Ct) values ranging from 27.6 to 37.9. The RT-PCR amplification of all eight AIV genes was performed for all four AIV-positive samples as previously described (12). Barcoded DNA libraries were prepared with the DNA Prep kit (Illumina, San Diego, USA), purified according to the manufacturer’s recommendations, and quantified using Qubit (Thermo Fisher Scientific). Pooled libraries were sequenced on the MiSeq platform (Illumina) using the 600-cycle V3 reagent kit (2 × 300 bp). Results obtained with NCBI Blastn (standard database of nucleotide collection (nr/nt)) using contigs obtained from *de novo* assemble (Geneious assembler) were used for template-guided assembly using map to reference (GenBank numbers: OR125242 to OR125247, OR265614 and OP888621) in Geneious Prime software version 2023.2.1 (https://www.geneious.com) with default parameters. The mean depth of coverage and the coverage distribution of each H6N2 virus segment varied from 2,667 to 16,026 and from 4.5% to 27%, respectively. Assembled genomes from Ab/BR/23 were uploaded to the GeneBank Database under the accession numbers PP333638 to PP333645. Maximum-likelihood (ML) tree was constructed using the RaxML v8.0 software (13) by a rapid bootstrapping of 1,000 trees with the GTR+Gamma model. Constructed trees were visualized using FigTree v1.4.

**Fig 1:**
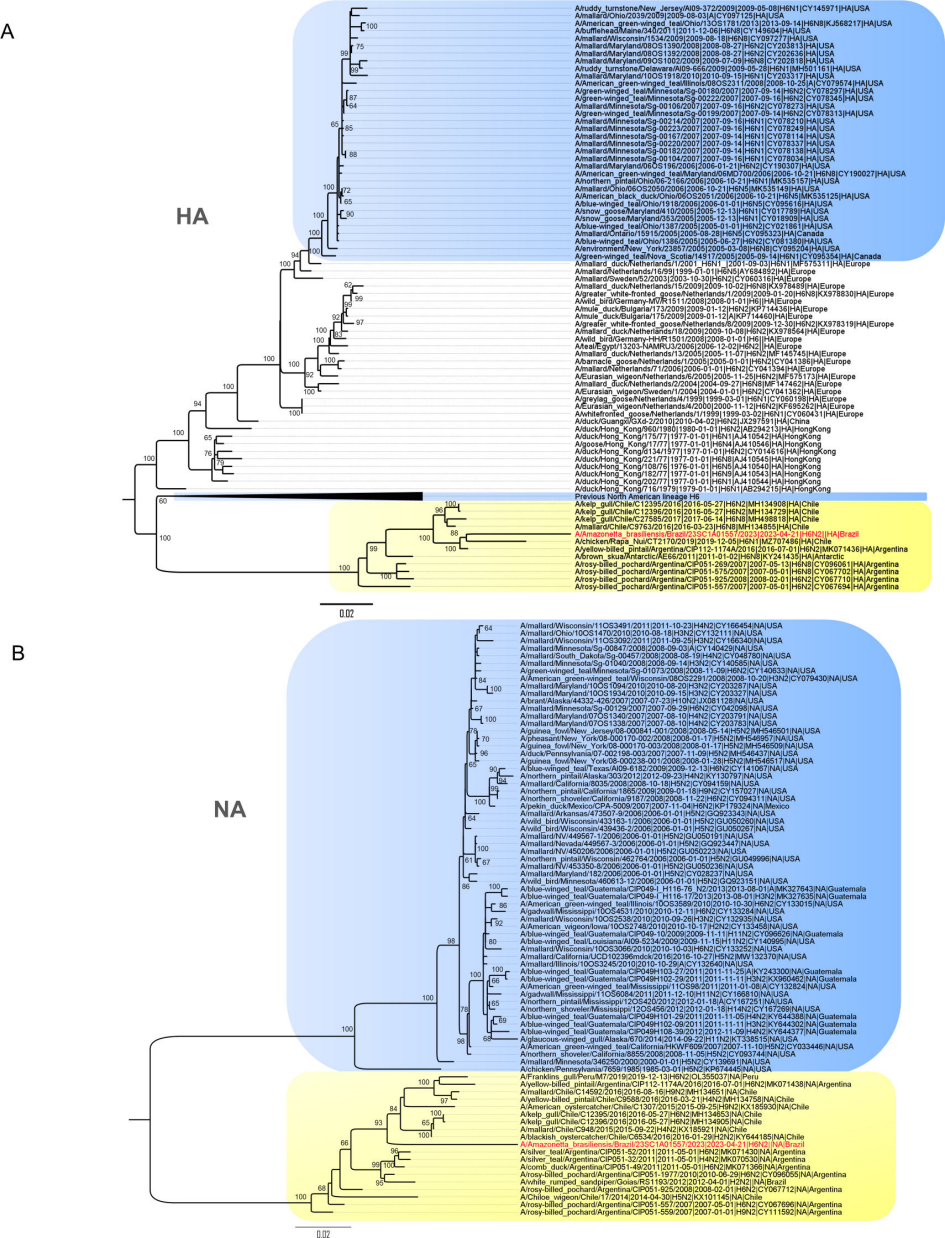
Maximum-likelihood tree constructed using RAxML v8.0 using the complete coding sequences of (**A**) hemagglutinin gene and (**B**) neuraminidase gene segments. Red taxa label indicates the virus sequenced in this study. Yellow shade highlights the South American cluster and blue shade highlights the North American cluster. Node labels indicate bootstrap value (%).

One complete genome sequence, with the lowest Ct, was obtained and named as A/Amazonetta brasiliensis/Brazil/23SC1A01557/2023 (H6N2) (hereafter, Ab/BR/23). Ab/Br/23 sequences had nucleotide identities varying from 94.4% to 98.3% when compared to other South American LPAIV sequences obtained from 2014 to 2016 (Table 1). Similarly, the phylogenetic analysis of all Ab/BR/23 genome segments, including HA and NA, shows that they clustered with the South American LPAIVs (5, 14), but with long branches on the ML trees, indicating the lack of surveillance and extensive circulation (Fig. 1). No evidence of reassortment was detected with the recent clade of 2.3.4.4 b H5N1 HPAV viruses (15).

**TABLE 1:** Nearest identities of Brazilian H6N2 low pathogenicity avian influenza virus (Ab/BR/23) from Brazil in 2023 and their GenBank accession numbers in the GenBank standard database (nr/nt) (as of 12 Sep 2023)

Isolate	Gene	Virus	GenBank accession no.	Sequence identity
Ab/BR/23	PB2	A/Chilean teal/Chile/12/2014 (A/H7N3)	KX101144	2189/2279 (96.05%)
	PB1	A/yellow-billed teal/Chile/C30879/2017 (A/H3N8)	MK163981	2200/2274 (96.75%)
	PA	A/yellow-billed pintail/Argentina/CIP112-1174A/2016 (A/H6N2)	MK071435	2083/2151 (96.84%)
	HA	A/yellow-billed pintail/Argentina/CIP112-1174A/2016 (A/H6N2)	MK071435	1632/1701 (95.94%)
	NP	A/Meleagris gallopavo/Valparaiso/22/2017 (A/H7N6)	MK424185	1494/1556 (96.02%)
	NA	A/blackish oystercatcher/Chile/C6534/2016 (A/H2N2)	KY644185	1332/1411 (94.40%)
	MP	A/yellow-billed teal/Chile/C30879/2017 (A/H3N8)	MK163981	965/982 (98.27%)
	NS	A/yellow-billed teal/Argentina/CIP112-1227/2016 (A/H4N6)	MK071456	814/836 (97.37%)

The present study highlights the need to increase the resolution and our understanding of the evolutionary history and genetic diversity of South American AIVs and their effects on the diversity of AIVs of the rest of the world.

## Data Availability

The datasets presented in this study can be found in online repositories. The names of the repository/repositories and accession number(s) can be found below: GeneBank Database under the accession numbers PP333638 to PP333645, and raw reads are publicly available at SRA file number: SRR28794995: https://www.ncbi.nlm.nih.gov/sra/PRJNA1103816.
